# Mutations in GTP Binding Protein Obg of *Mycoplasma synoviae* Vaccine Strain MS-H: Implications in Temperature-Sensitivity Phenotype

**DOI:** 10.1371/journal.pone.0073954

**Published:** 2013-09-17

**Authors:** Muhammad A. Shahid, Philip F. Markham, John F. Markham, Marc S. Marenda, Amir H. Noormohammadi

**Affiliations:** 1 Faculty of Veterinary Science, The University of Melbourne, Werribee, Victoria, Australia; 2 Asia-Pacific Centre for Animal Health, Faculty of Veterinary Science, The University of Melbourne, Parkville, Victoria, Australia; 3 National ICT Australia (NICTA) Victoria Research Laboratory, Department of Electrical and Electronic Engineering, School of Engineering, The University of Melbourne, Melbourne, Victoria, Australia; University Paris South, France

## Abstract

*Mycoplasma synoviae* strain MS-H, developed by chemical mutagenesis of the Australian field strain 86079/7NS, is a live temperature-sensitive (*ts* ^+^) vaccine used for control of *M. synoviae* infection in poultry worldwide. Genetic basis of temperature sensitivity and attenuation of MS-H has not been revealed thus far. Comparison of the complete genome sequence of MS-H, its parent strain 86079/7NS and two non-temperature sensitive (*ts* ^–^) reisolates of MS-H revealed a mutation in a highly conserved domain of GTP binding protein Obg of MS-H, with reversion in *ts* ^–^ MS-H reisolates. Nucleotide change from G to A at position 369 of the *obg* gene resulted in an alteration of glycine to arginine at position 123 in Obg fold. Further analysis of the complete *obg* gene sequence in several MS-H reisolates revealed that a Gly123Arg substitution was associated with alteration in temperature sensitivity phenotype of MS-H. A second mutation, C to T at position 629, in *obg* gene was found in some of the MS-H reisolates and appeared to suppress the effects of the Gly123Arg substitution. *In silico* analysis of point mutations revealed that Gly123Arg has highly destabilizing effect on the MS-H Obg structure that can potentially abolish its biological functions *in vivo* especially at non-permissive temperature. Findings of this study implicate Obg alteration (Gly123Arg) as one of the possible causes of MS-H attenuation/temperature sensitivity and warrant further investigations into exploring the role of Obg-like proteins, an evolutionarily conserved protein from human to bacteria, in the biology of mycoplasmas.

## Introduction

Obg is one of the GTP (Guanosine-5′-triphosphate) binding proteins belonging to GTPase superfamily [1]–[3]. GTP binding proteins are found in all living organisms ranging from human to bacteria and are involved in the essential cellular processes such as signal transduction, protein synthesis, membrane trafficking and cell proliferation. Obg was originally identified in *Bacillus subtilis,* encoded by a gene with a GTP binding domain located downstream of a sporulation stage 0 gene *spo0B*; therefore, the name Obg originated from *spo0B*-associated GTP binding protein [4]. The Obg subfamily along with four other subfamilies (DRG, YyaF/YchF, Ygr210, and NOG1) belongs to the OBG family which includes highly conserved bacterial and eukaryotic GTP binding proteins [5].

Bacterial members of the Obg subfamily are Obg from *B. subtilis*, *Streptomyces griseus* and *S. coelicolor*, CgtA proteins from *Caulobacter crescentus*, *Escherichia coli* (*E. coli* CgtA is also called ObgE) and *Vibrio harveyi*, and YhbZ from *Haemophilus influenzae* [6]. The Obg subfamily GTPases are involved in diverse essential cellular functions including cell growth, morphological differentiation, DNA replication [7], chromosome segregation [8], early steps of sporulation [9], ribosome assembly [10] and stress dependent activation of σ^B^ transcription factor that controls a cellular response to environmental stress [11], [12]. The exact mechanism of Obg function is unknown but bacterial Obg has been found associated with ribosome(s), specifically bound to ribosomal protein L13 and therefore seems to be involved in ribosome biogenesis, maturation and assembly [13]–[19], presumably as a rRNA/ribosomal protein folding chaperone or scaffolding protein [16]. Consequently, Obg proteins have been considered essential for cell viability in various bacteria [3] including *Mycoplasma genitalium* [20], [21].

The three dimensional (3D) structure of C-terminally truncated form (residues 1–342) of *B. subtilis* Obg [22] and full-length form (residues 1–416) of *Thermus thermophilus* Obg [23] has been determined. The full-length *T*. *thermophilus* Obg is found to contain N-terminal, GTP-binding (G domain) and C-terminal domains. The N-terminal domain has an Obg fold and the G domain contains a Ras-like GTPase fold. These folds share high levels of amino acid similarity with those of the C-terminal domain of the truncated form of *B. subtilis* Obg. The C-terminal domain of *T*. *thermophilus* Obg has a novel Obg-C-terminal (OCT) fold which, on the basis of amino acid identity, is likely to be found in *B. subtilis* Obg as well [23]. This C-terminal domain is also called TGS domain, named for stress response related protein families (ThrRS, GTPase, and SpoT) in which it is found. Structural observation of ppGpp nucleotide, a specific nucleotide synthesized extensively in amino acid-starved cells serving as starvation alarmone and global regulator of gene expression, within the active site of *B. subtilis* Obg suggests a possible role of Obg function through ppGpp modulation. It has been speculated that Obg protein may have evolved to recognize ppGpp in response to adverse cellular environment [22].

The Obg fold in *B. subtilis* Obg contains 26 glycine residues organized into linear sequence motifs, 21 of which are conserved between Obg subfamily members. Structural analysis revealed that the glycine-rich motifs are comprised of 6 left-handed type-II helices (a, b, c, d, e, and f),tightly packed together in pairs (a–b, c–d, and e–f) in both parallel and antiparallel fashion. The helices are arranged to form a complex main-chain hydrogen bonding pattern, with each helix making at least one main-chain hydrogen bonding interaction with at least two other helices [22]. Evidence for the putative functional role of Obg fold stemmed from the isolation of temperature-sensitive (*ts* ^+^) *obg* alleles of *B. subtilis* [7] and *E. coli* [24] with mutations in the glycine-rich domain. These studies concluded that integrity of Obg fold is required for the *in vivo* function of Obg [22]. The G domain contains GTPase superfamily consensus motifs (G1, G2, G3, G4 and G5) and putative switch-I and switch-II elements. The switch elements presumably mediate interaction between Obg fold and G domain and perhaps are involved in a feedback mechanism between two domains in response to GTP/GDP binding [22]. Previously, substitution of serine with proline at position 314 in the G domain (G5 motif) of *E. coli* Obg rendered the cells as *ts* ^+^ [16], [24].

Temperature-sensitive (*ts*) mutants of viruses [25], [26] and bacteria [27]–[29] have been used as vaccine candidates and a large number of such mutants have been studied to define gene functions [30]. However, it is not precisely known whether temperature sensitivity is the cause of attenuation or just a coincidental phenotype in bacterial *ts* vaccines. In our previous study, the *ts* phenotype of 23 *Mycoplasma synoviae* strains/isolates, including various reisolates of the MS-H vaccine, was determined [31]. In this study, comparative genomic analysis of *ts* ^+^ *M. synoviae* vaccine strain MS-H with its non-temperature-sensitive (*ts* ^–^) parent strain 86079/7NS and two *ts* ^–^ reisolates of MS-H (MS-H^4^ and MS-H^5^) revealed a single mutation (G→A) in *obg* associated with *ts* ^+^ phenotype. This mutation resulted in an amino acid change from glycine (Gly) to arginine (Arg) at position 123 (Gly123Arg) of the Obg fold. Single nucleotide polymorphism (SNP) resulting in amino acid substitution may have an effect on the structure, thereby on the function of the protein [32], [33]. Therefore, we further investigated the correlation of *ts* phenotype with Obg mutation (Gly123Arg) by analysis of the complete *obg* sequence from several *M. synoviae* strains/isolates. We also conducted theoretical studies on *M. synoviae* Obg homology models to predict the effect of Obg mutations on the structure-function relationship.

## Materials and Methods

### *M. synoviae* Strains Used in this Study

*M. synoviae* strains used in this study included vaccine strain MS-H, its parent/wild-type strain 86079/7NS and 20 reisolates of MS-H (including MS-H^4^ and MS-H^5^), recovered from MS-H vaccinated flocks, with restriction fragment length polymorphism patterns identical to MS-H [34]. The origin/source and *ts* phenotype of these strains have been described in our previous study [31] and included in this paper as Table S1.

### SOLiD™ Sequencing and Assembly of *M. synoviae* Strains Genomes

Phenol-chloroform extracted genomic DNA from MS-H, 86079/7NS, MS-H^4^ and MS-H^5^ was subjected to DNA library preparation for ligation-mediated sequencing technology (SOLiD™ 3 system; Applied Biosystems, Foster City, USA) as recommended by the manufacturer. Briefly, 1 µg of *M. synoviae* DNA was sheared and resulting fragments were ligated to P1 and P2 adaptors (SOLiD™ Library Oligos Kit 1; Applied Biosystems, Foster City, USA). Ligated fragments were size-selected to an average length of 170 bp followed by amplification for 10 cycles. Approximately 50 pg/µl of this fragment library was added to 80 million beads emulsion. The library was sequenced using a flow cell divided into 8 segments. The resulting reads (∼ 50 nucleotides in length) were mapped to bacterial reference genome with two mismatches per read. Filtered reads of MS-H, 86079/7NS, MS-H^4^ and MS-H^5^ were assembled with Corona Lite package v4.2 (Applied Biosystems) using *M. synoviae* strain MS-53 as reference (GenBank accession no. AE017245). CodonCode Aligner v.3.7.1 was used to call SNPs among study strains keeping 86079/7NS as reference genotype.

### Genomic DNA Extraction from *M. synoviae* Strains/Isolates for Complete *obg* Sequence

To extract genomic DNA, *M. synoviae* strains/isolates were cultured in 1 ml of Mycoplasma broth at 37°C overnight. The cells were harvested by centrifugation at 14,000×*g* for 3 min and the cell pellet resuspended in 500 µl RLT lysis buffer (Qiagen, Chadstone, Australia) containing 1% of 2-ß-mercaptoethanol and incubated at 4°C overnight. Following incubation, 15 µl of Qiaex II (Qiagen) and 300 µl of 70% ethanol were added, mixed and the suspension loaded onto a multispin MSK-11 column (Axygen, Union City, USA). The column was centrifuged for 30 sec at 10,000×*g* and the flow-through discarded. The column was washed once with 600 µl of RW1 buffer (Qiagen) and twice with 500 µl of RPE buffer (Qiagen) by centrifugation for 30 sec at 10,000×*g* and then dried by centrifugation for 90 sec at 14,000×*g*. Finally, 50 µl of DNAse free water was added to the column, incubated for 5 min at room temperature, and then genomic DNA was eluted by centrifugation at 10,000×*g* for 60 sec.

### Determination of the Complete *obg* Gene Sequence of *M. synoviae* Strains/Isolates

The complete *obg* coding DNA sequence (CDS) for MS-H and 86079/7NS was obtained using SOLiD and Illumina sequencing technologies. The complete *obg* CDS for twenty MS-H reisolates was amplified using oligonucleotide primers obg-comF (5′-CTG AAG AAC AAA CAG TTA ATG G-3′) and obg-comR2 (5′-AA TAG CAC CAA GAT AAT TTC C-3′). For PCR, a 50 µl PCR reaction mixture contained 2 µl of genomic DNA, 8 µl of each of 1.25 mM dNTP (Promega, Alexandria, Australia), 4 µl of 25 mM MgCl_2_, 1 µl of each 25 µM forward and reverse primers, 0.2 µl of Taq DNA polymerase (Promega), 10 µl of 5× GoTaq flexi green buffer (Promega) and 23.8 µl of nuclease free water. The PCR reaction was subjected to one cycle of denaturation at 95°C for 2 min followed by 45 cycles of 20 sec at 95°C, 20 sec at 52°C and 2 min at 72°C, followed by a final extension of 5 min at 72°C. All PCR reactions were carried out using an *i*Cycler thermocycler (Biorad, Gladesville, Australia). After PCR, 5 µl of amplified DNA was loaded onto a 0.7% agarose gel containing 5 ml of 10, 000× GelRed™ (Biotium, Hayward, USA) and 0.5× TBE (44.5 mM Tris-HCl, 44.5 mM Boric acid, 1.9 mM EDTA, pH 8.0). The gel was subjected to electrophoresis at 80 V for 60 min and DNA bands visualized by UV transillumination using a Gel Logic 1500 Imaging System (Kodak, New Haven, USA).

Amplicons were purified using Wizard® SV Gel and PCR Clean-Up system (Promega), eluted in 30 µl nuclease free water and subjected to automated DNA sequencing (BigDye® Terminator v.3.1 chemistry; Applied Biosystems) in both directions using external primers as described above and two internal primers, obg-F (5′-GTT GAT AAA GGT GGA CCA G-3′) and obg-R (5′-TTA GTG CAG ATA TCT CAA TG-3′). Nucleotide sequencing reads for each of the 20 *M. synoviae* strains/isolates were edited using Geneious® Pro software package v.5.5.6 (Biomatters, New Zealand) [35] and reads with no ambiguities were assembled and the consensus sequence representing the complete *obg* CDS was obtained using the Contig assembly function of the Geneious®. Nucleotide sequences of complete *obg* CDS for *M. synoviae* strains 86079/7NS, MS-H, 93198/1-24b and 94036/2-2a (as representatives of four different types of sequence) have been deposited in GenBank under accession numbers KC990840, KC990837, KC990838 and KC990839, respectively.

### Homology Modeling of Obg Proteins

The SWISS-MODEL workspace (http://swissmodel.expasy.org/workspace/) [36] was used for homology modelling of translated Obg protein sequences deduced from the complete *obg* nucleotide sequences. A template search was conducted using template identification tools available at SWISS-MODEL workspace employing BLAST, PSI-BLAST and HMM (Hidden Markov Model) based HHSearch. The *B. subtilis* and *T. thermophilus* Obg proteins (PDB ID: 1 LNZ and PDB ID: 1 UDX respectively) were selected on the basis of percentage amino acid identity and coverage of the target sequences respectively. Crystal structure for *B. subtilis* Obg protein has been determined for only the N-terminal 342 residues (total length 428) comprising only Obg and G domains [22] whereas the crystal structure of the complete Obg protein (416 residues) of *T. thermophilus* has been determined and includes the C-terminal (OCT or TGS) domain. The automated mode of the SWISS-MODEL workspace was used to obtain the homology models of *M. synoviae* Obg proteins. Conserved sequence motifs including type-II helices, GTP binding motifs G1–G5, and secondary structures (either α-helices or β-strands) were also identified using annotations of PDB entry 1 UDX. The remaining amino acid sequences were examined following the multiple alignment of *M. synoviae* Obg protein sequences with other Obg subfamily members to improve the quality of the model using alignment mode of SWISS-MODEL workspace. The final alignment between target (86079/7NS Obg) and template showed an overall amino acid identity of 40% (*B. subtilis* Obg as template) and 36% (*T. thermophilus* Obg as template). Higher amino acid identity in the conserved Obg and GTP binding domains, 46% with *B. subtilis* Obg and 40% with *T. thermophilus* Obg, indicated that these 3D models were the best possible templates for the construction of a relatively reliable 3D model of *M. synoviae* Obg proteins.

### Quality Assessment of Predicted 3D Structures

Amino acid sequence identity is considered as the first check in protein model quality assessment. Therefore, templates *T. thermophilus* Obg and *B. subtilis* Obg, that showed the highest percentage identity with *M. synoviae* Obg, were selected. Furthermore stereochemistry check was performed using the program PROCHECK [37] and Global model quality was assessed using DFIRE [38] and QMEAN6 composite scoring function [39]. Other programs used for structure quality assessment were Verify-3D [40] and ERRAT [41]. DFIRE, PROCHECK and QMEAN6 quality assessments were performed using the structure assessment module available at SWISS-MODEL workspace (http://swissmodel.expasy.org/workspace/) while Verify-3D and ERRAT quality assessments were performed using Structural Analysis and Verification Server (http://nihserver.mbi.ucla.edu/SAVS/).

### Prediction of Impact of Mutations on *M. synoviae* Obg Stability

Effect of amino acid substitutions on the stability of *M. synoviae* Obg was assessed by SDM (Site directed mutator) program [42] available online (http://mordred.bioc.cam.ac.uk/~sdm/sdm.php). Predicted 3D models of 86079/7NS, MS-H, 93198/1-24b and 94036/2-2a Obg proteins were used in the SDM program to examine the effects of mutations on the folding stability of protein. SDM program detects the effect of mutation between two given protein models if differing at only one residue [42]. Therefore, to predict the effect of mutations in *M. synoviae* Obg, e.g., Gly123Arg, 86079/7NS was used a wild-type and MS-H as mutant. For other mutations, *M. synoviae* strains/isolates were used as wild-type and mutant accordingly.

## Results

### Identification and Selection of SNPs from *M. synoviae* Genomic Sequences

A total number of 45 SNPs were detected when partially sequenced genomes of MS-H, 86079/7NS, MS-H^4^ and MS-H^5^ were compared. Only those SNPs which were found in MS-H and absent in MS-H^4^ and MS-H^5^ were targeted in this study. Four SNPs of such type were found in MS-H and therefore were considered to have potential involvement in *ts* phenotype of MS-H. Note that the MS-H is *ts* ^+^ while 86079/7NS, MS-H^4^ and MS-H^5^ are *ts* ^–^. Three of these SNPs were within the coding sequence of the genes *nagB*, *cls* and *pepO* (A→G, A→G and G→A at positions 216554, 445923 and 584515, respectively, according to GenBank accession no. AE017245) but all found to be of synonymous nature and were therefore not pursued any further. The remaining SNP (G→A, at position 181212 according to GenBank accession no. AE017245), located in the gene *obg*, was of non-synonymous nature and was further examined by Sanger sequencing (BDT version 3.1; Applied Biosystems) using primers described in materials and methods. Mutations in Obg of both Gram-positive and Gram-negative bacteria have been identified in association with *ts* ^+^ [7], [16], [24]. Therefore, *M. synoviae* Obg was selected for further analysis towards a potential cause of MS-H temperature-sensitivity and/or attenuation.

### Glycine to Arginine Substitution at Position 123 of Obg Associated with *ts* ^+^ Phenotype in most *ts* ^+^ MS-H Reisolates

**Figure 1:**
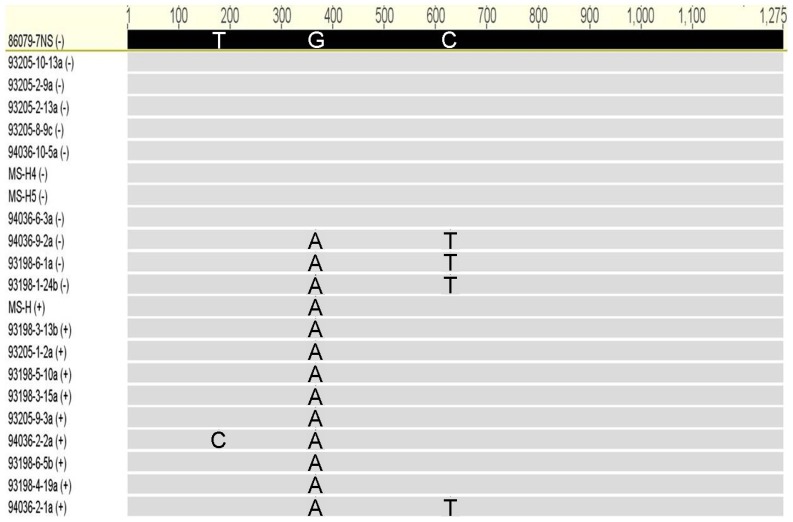
Alignment of complete *obg* coding DNA sequences from *M. synoviae* MS-H, its parent strain 86079/7NS and 20 of its reisolates. Position of nucleotides are shown above. Nucleotide differences to 86079/7NS at residue positions 178 (T→C), 367 (G→A), and 629 (C→T) are indicated. Temperature sensitivity phenotypes have also been given in brackets as − or + for non-temperature-sensitive and temperature-sensitive respectively.

**Figure 2:**
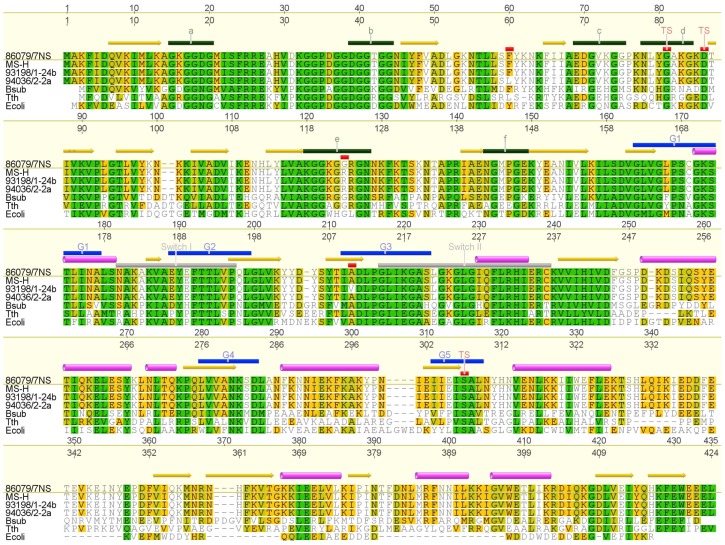
Alignment of Obg amino acid sequences. Alignment was performed using Clustal W (Blosum 62 scoring matrix). Obg from vaccine MS-H, MS-H reisolates 93198/1-24b, 94036/2-2a, *B. subtilis* (Bsub), *T. thermophilus* (Tth) and *E. coli* (Ecoli) were compared to that of *M. synoviae* 86079/7NS as reference. Numbering is done with respect to consensus (upper) and reference (lower). The nucleotide binding motifs (G1–G5) are indicated by blue bars above the sequence. The secondary structures including alpha helices are shown using pink barrels, beta strands using yellow arrows, switch elements using gray bars and type-II helices a–f using green bars. These predictions were made according to the crystal structure of *T. thermophilus* Obg [23]. Amino acid changes due to SNPs in the Obg of the MS-H and its reisolates are depicted as red bars above the amino acid change while point mutations responsible for temperature-sensitivity in *B. subtilis* and *E. coli* are indicated as red bars with TS annotation. All annotations were made using Geneious™ software version 5.6.2 [35].

Alignment of complete *obg* nucleotide sequences from 86079/7NS (used as reference genotype), MS-H and 20 MS-H reisolates revealed nucleotide polymorphism at nucleotide positions 178, 367 and 629 (Figure 1) all of which were found to produce non-synonymous amino acid changes (Figure 2). Alignment of the deduced amino acid sequences from *ts* ^–^86079/7NS and *ts* ^+^ MS-H *obg* showed only one amino acid difference at position 123 with the substitution of a glycine residue in 86079/7NS to an arginine (Gly123Arg) in MS-H (Figure 2). Eight out of twenty MS-H reisolates had identical amino acid sequences to, and showed a *ts* phenotype consistent with, that of 86079/7NS (*ts* ^–^). Seven reisolates had amino acid sequence and *ts* phenotype that were identical to those of MS-H (*ts* ^+^). Another 4 reisolates had identical sequence to MS-H except each had an amino acid substitution at position 210 (Ala210Val), three of these isolates had *ts* phenotype consistent with that 86079/7NS whilst one had a *ts* phenotype consistent with MS-H (*ts* ^+^). Also a single *ts* ^+^ isolate had an identical amino acid sequence to MS-H except it had a substitution of a phenylalanine to leucine at position 60 (Phe60Leu). The amino acid variation Gly123Arg was mapped within the highly conserved Obg domain (more precisely in one of the glycine-rich loops also known as type-II helices) of the protein (Figure 2). The amino acid substitution Ala210Val was mapped in the beta strand 13 (β13) which constitutes part of the putative GTP binding motif G3. The substitution Phe60Leu was also mapped within the Obg fold of the protein (Figure 2).

### Homology Models of Reliable Quality Determined for *M. synoviae* Obg Proteins

**Figure 3:**
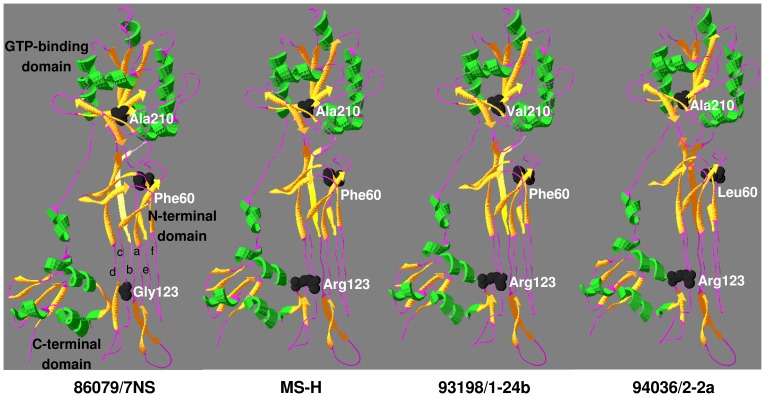
Ribbon presentation of Obg from M. synoviae strains/isolates 86079/7NS (ts^–^), MS-H (ts^+^), 93198/1-24b (ts^–^) and 94036/2-2a (ts^+^). Substitution of glycine to arginine at position 123 of Obg (within the Obg-fold) is associated with ts^+^ phenotype while an additional substitution of alanine to valine at position 210 (within the GTP-binding domain) appears to restore the wild-type ts^–^ phenotype. Phenylalanine to leucine substitution at position 60 has neutral effect on Obg. T. thermophilus Obg (PDB ID: 1 UDX) was used as template in homology modeling. Location of mutated amino acids have been highlighted for comparison purpose. 86079/7NS Obg domains are labeled and type-II helices are lettered in lowercase. Images were produced using Swiss-PdbViewer v4.04 [54].

**Figure 4:**
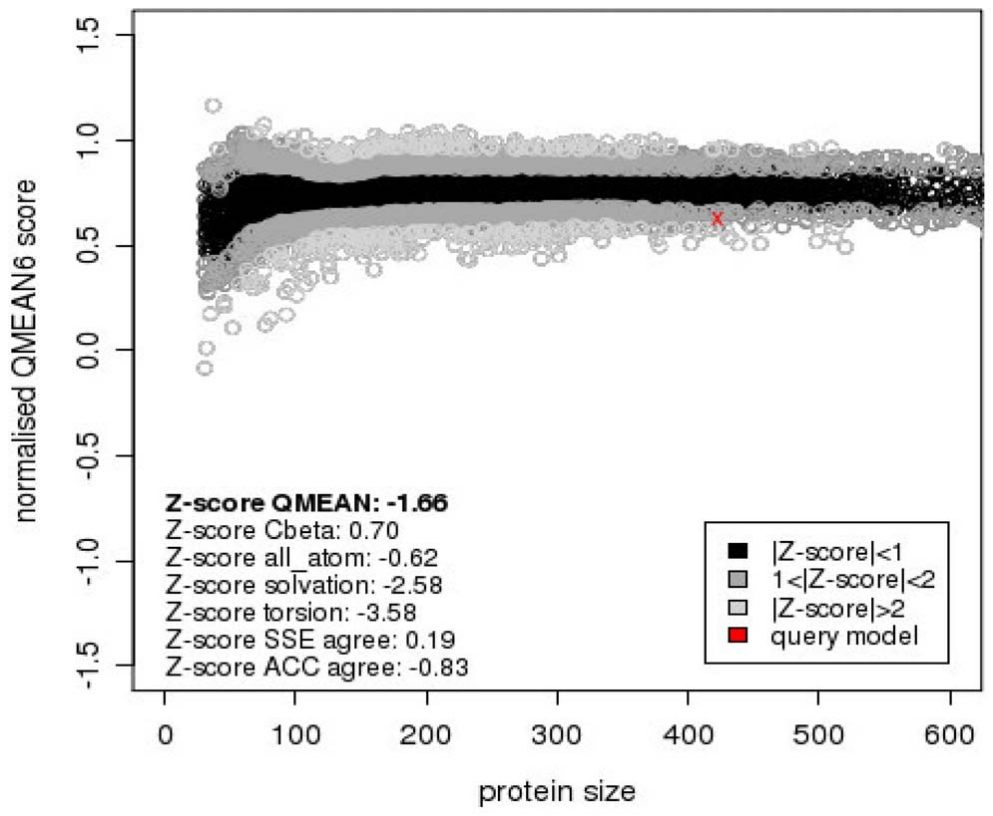
Assessment of quality of *M. synoviae* strain 86079/7NS Obg homology model by QMEAN6 scoring function. The model was of high quality and predicted the impact of Gly123Arg substitution on the function of *M. synoviae* Obg. Comparison of the predicted 3D model of 86079/7NS Obg (using *T. thermophilus* Obg as template) was made with a non-redundant set of high resolution structures available in PDB in terms of QMEAN6 and QMEAN Z-scores. Predicted QMEAN6 and Z-scores, shown with red cross, of 86079/7NS Obg model were within the limits of good quality structures.

**Figure 5:**
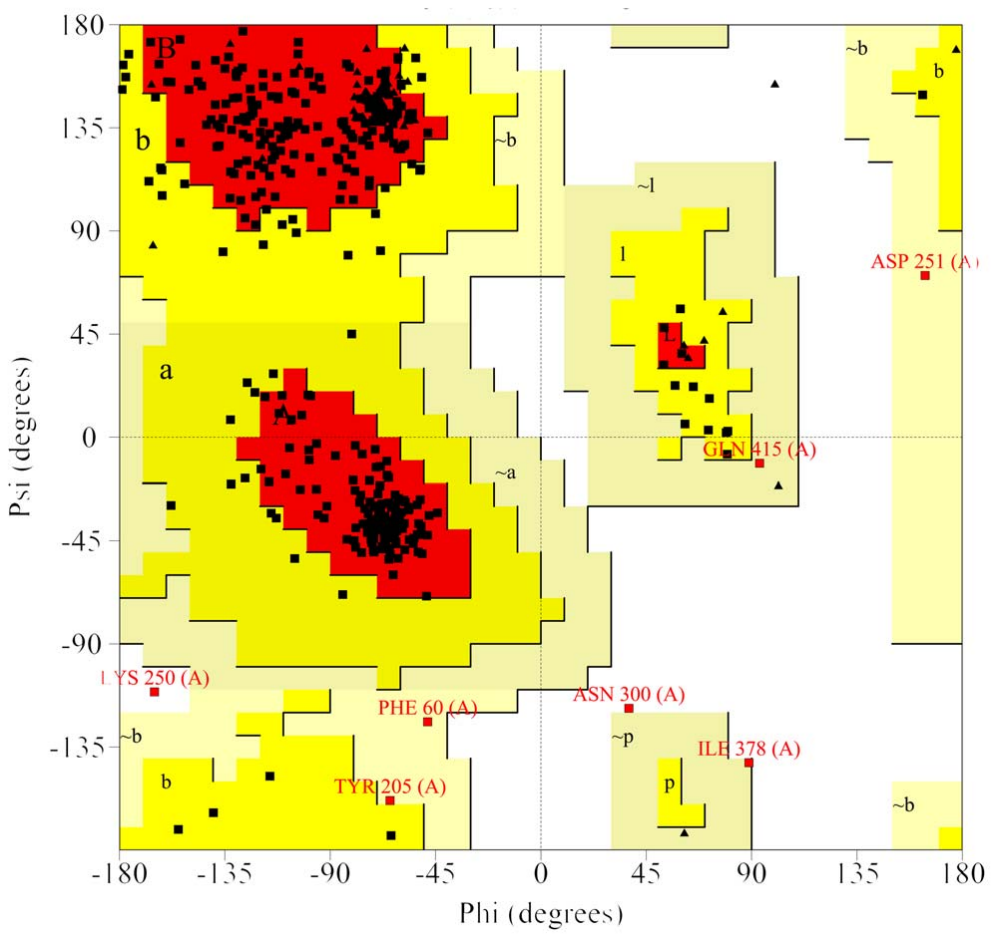
Quality assessment of *M. synoviae* strain 86079/7NS Obg homology model by Ramachandran plot. The model was of high quality and predicted the impact of Gly123Arg substitution on the function of *M. synoviae* Obg. Ramachandran plot obtained using PROCHECK program examining the stereochemical quality of 86079/7NS Obg model (*T. thermophilus* Obg as template). Factors considered were distribution of residues in most favored regions (red shaded, 84.8%), additional allowed regions (yellow shaded, 13.3%), generously allowed (khaki shaded, 1.4%) and disallowed regions (white shaded, 0.5%).

Predicted 3D models for Obg proteins of 86079/7NS, MS-H, 93198/1-24b (representative of 4 reisolates with amino acid substitution Ala210Val) and 94036/2-2a are shown in Figure 3. Quality assessment data of a 3D model for 86079/7NS Obg (referred as target hereafter) is shown in the results described below. The QMEAN6 raw and Z-score of 0.628 and –1.66 respectively were obtained for the target when *T. thermophilus* Obg (PDB ID: 1UDX) was used as template (Figure 4). These scores were 0.566 and –2.42 respectively when *B. subtilis* Obg (PDB ID: 1LNZ) was used as template (graph not shown). DFIRE energy scores when *T. thermophilus* Obg and *B. subtilis* Obg were used as templates were –499.68 and –384.84 respectively. Stereochemical quality of each residue in the target was determined by Ramachandran plot (shows percentage of residues in allowed regions) (Figure 5). The stereochemical quality scores were 84.8% and 77.5% when *T. thermophilus* Obg and *B. subtilis* Obg were used as template respectively, suggesting a model of good quality. Verify-3D analysis showed 78.25 and 82.09% residues with a score of >0.2 when *T. thermophilus* Obg and *B. subtilis* Obg were used as templates respectively. ERRAT overall quality factor was 58.19 and 56.87 when *T. thermophilus* Obg and *B. subtilis* Obg were used as templates respectively, indicating a target protein model of good quality.

### Predicted Instability of *M. synoviae* Obg Protein caused by Gly123Arg Substitution

3D models of variants of *M. synoviae* Obg proteins were generated using the SWISS-MODEL and the PDB output format files were used in the SDM program to predict the effect of point mutations on Obg stability and/or function. The substitution Gly123Arg in MS-H Obg was highly destabilizing with delta-delta G value of –2.50 and –2.12 when *B. subtilis* Obg and *T. thermophilus* Obg were used as templates respectively. Therefore, this mutation had the potential to affect the structure of the protein and result in malfunction or abrogation of the function. The other amino acid substitutions Ala210Val and Phe60Leu were predicted to be either stabilizing or neutral respectively, with delta-delta G value of 1.02 for Ala210Val (*B. subtilis* Obg and *T. thermophilus* Obg as template) and –0.17 and 1.08 for Phe60Leu when *B. subtilis* and *T. thermophilus* Obg were used as templates respectively (Table 1).

**Table 1:** Prediction of stability after amino acid substitutions in the 3D structures of *M. synoviae* Obg proteins using SDM program [42].

Mutation	Amino acid number	*T. thermophilus* Obg as template			*B. subtilis* Obg as template		
		aΔΔG (kcal/mol)	bSolvent accessibility (%)	Effect on stability	aΔΔG (kcal/mol)	bSolvent accessibility (%)	Effect on stability
Gly→Arg	123	–2.12	0.9–2.5 (buried in wild-type and mutant)	Highly destabilizing and causemalfunction	–2.50	5–28.1% (partially accessible inmutant)	Highly destabilizing and causemalfunction
Ala→Val	210	1.02	4.7-1% (buried in wild-type andmutant)	Stabilizing and no malfunction	1.02	0 (buried in wild-type and mutant)	Stabilizing and no malfunction
Phe→Leu	60	1.08	7–7.7 (buried in wild-type andmutant)	Stabilizing and no malfunction	–0.17	5.9–12.6 (buried in wild-type andmutant)	Neutral

a Negative ΔΔG is destabilizing and <–2 causes malfunction, while positive is stabilizing and >+2 causes malfunction [42].

b Solvent accessibility of amino acid sidechain indicated from wild-type to mutant; <17% is inaccessible/buried, 17–43% is partially accessible and >43% is accessible [42].

## Discussion

Computer modeling has been used to predict the effects of SNPs on the structure of proteins but this requires a previously determined 3D structure of protein(s) with high homology to the protein under investigation. In this process, proper alignment of the target and template and the degree of the similarity is important for accuracy of the predicted model. Homology modeling can still be used if the target-template identity is >25% but reliability decreases with decrease in identity [43]. Overall amino acid sequence identity between *M. synoviae* Obg and *B. subtilis* Obg was 40%, but identity over both Obg and GTP binding domains was 46%. For *B. subtilis* Obg, 3D structure of only these two domains (1–342 residues; 2.60 A resolution) has been elucidated experimentally [22]. For *T. thermophilus* however, the complete Obg crystal structure (1–416 residues; 2.07 A) has been determined [23]. The overall and partial (conserved Obg and GTP-binding domains) amino acid sequence identity of *T. thermophilus* Obg with *M. synoviae* Obg was 36% and 40% respectively. Therefore, in this study, both PDB entries *B. subtilis* Obg (1 LNZ) and *T. thermophilus* Obg (1 UDX), were used as template during homology modelling to augment reliability of the predicted 3D models for *M. synoviae* Obg proteins.

There is no single method available to consistently and accurately predict the errors in a protein structure or a homology model. Therefore, multiple methods are often used to assess protein structure quality either predicted by homology modeling or experimentally resolved crystal structures [44]. In this study, quality assessments were achieved using various programs including DFIRE, QMEAN6, PROCHECK, Verify-3D and ERRAT. DFIRE is used to assess non-bonded atomic interactions in the protein with a model of lower energy indicating a structure closer to native conformation [38]. DFIRE energy of the target in this study (86079/7NS Obg) was –499.68 and –384.84 using *T. thermophilus* and *B. subtilis* Obg as templates respectively, indicating the *T. thermophilus* Obg based model was more reliable. QMEAN6 is a linear combination of 6 structural descriptors using statistical potentials for estimation of global and local model quality. QMEAN6 raw score ranges from 0 to 1 (1 being the best model). The QMEAN Z-score represents measure of the absolute quality of a model by providing an estimate of the ‘degree of nativeness’ of structural features observed in a model and by describing the likelihood that a given model is of comparable quality to experimental structures. Models of low quality are expected to have strongly negative QMEAN Z-scores [39], [45], [46]. The average Z-score of high-resolution reference structures are 0. For target Obg predicted models, the QMEAN6 raw scores of 0.628 (*T. thermophilus* Obg as template) and 0.566 (*B. subtilis* Obg as template) and Z-scores of –1.658 (*T. thermophilus* Obg as template) and –2.417 (*B. subtilis* Obg as template) were obtained indicating that the *T. thermophilus* Obg based model was better suited. The Ramachandran plot, generated by PROCHECK, indicated 84.8% (*T. thermophilus* Obg as template) and 77.5% (*B. subtilis* Obg as template) of residues in the allowed regions. In this program, good quality models often generate higher than 90% residues within the allowed regions. Although these scores both fell under a level considered good quality for a model, the *T. thermophilus* Obg based model was a comparatively better predictor for *M. synoviae* Obg. Verify-3D uses energetic and empirical methods to produce averaged data points for each residue to evaluate the quality of protein structures. In this scoring system, if >80% residues have a score >0.2 the protein structure is then considered to be of good quality [40]. In this study when 86079/7NS Obg was used as target, 78.25% (*T. thermophilus* Obg as template) and 82.09% (*B. subtilis* Obg as template) of the residues had score of >0.2, indicating models of good quality. In this study, ERRAT was also used to evaluate the quality of *M. synovie* Obg model. ERRAT is the so-called “overall quality factor” for non-bonded atomic interactions, with higher scores indicating higher quality. Normally the acceptable score is >50 [41]. In this study, target Obg model had ERRAT scores of 58.19 when *T. thermophilus* Obg was used as template, and 56.87 when *B. subtilis* Obg was used as template, both reflecting good models of prediction.

The potential effects of SNPs on protein stability were predicted using the software SDM which is a statistical potential energy function [47]. It uses environment-specific amino acid substitution frequencies within homologous protein families to calculate a stability score analogous to the free energy difference between wild-type and mutant protein. This method has been found to perform more reliably than other published methods in classifying the mutations as stabilizing and destabilizing [32], [42]. Furthermore, it was found to have higher accuracy in predicting whether protein mutants had an association with disease or malfunction [32], [48]. In general, the predicted *M. synoviae* Obg 3D models based on *B. subtilis* and *T. thermophilus* Obgs as templates, were in agreement in this study. In case of the Gly123Arg mutation, the solvent accessibility in the mutant was predicted to be higher in *B. subtilis* Obg based model than that based on *T. thermophilus* Obg. This is likely to be due to the higher amino acid identity of *B. subtilis* Obg with *M. synoviae* Obg in their Obg and GTP domains. Highly destabilizing effect of this mutation and its correlation with *ts* ^+^ phenotype (Figure 1) suggests that mutation detected here in Obg of *M. synoviae* may be responsible for alteration of temperature-sensitive phenotype in this organism. This hypothesis is supported by the highly conserved nature of this glycine residue in type-II helix e (Figure 2). Amino acid substitution Ala210Val in the GTP binding motif G3, which constitutes part of the switch element II (Figure 2), with a predicted stabilizing effect might be responsible for restoring the *ts* ^–^ phenotype in these reisolates (Figure 1). The putative GTPase switch elements mediate interaction between Obg fold and GTP binding domain suggesting a possible feedback mechanism between the two domains in response to GTP/GDP binding [22]. Therefore, it is possible that the mutation Ala210Val could provide stability for the Obg structure which is otherwise impaired by Gly123Arg mutation. It is notable that among the GTPase consensus motifs; G1, G3 and G4 have been found to bind and hydrolyse GTP, and also interact with cofactor Mg^2+^ [2]. Therefore the mutation Ala210Val, which is mapped in the G3 motif, may have a significant effect on the structure and function of mutant Obg. The observation that the *ts* ^+^ reisolate 94036/2-1a with a similar Ala210Val mutation to that of 93198/1-24b but *ts* ^+^ phenotype may reflect involvement of factors other than Obg, such as presence of extragenic suppressors of Obg such as tRNAs [49]–[51] in temperature sensitivity of MS-H.

The *obg* gene has been shown to be essential for viability in *B. subtilis*, *Vibrio cholerae*, *V. harveyi*, *S. coelicolor*, *C. crescentus*, *E. coli*, *Streptococcus pneumoniae*, *Streptococcus aureus*, *H. influenzae* [3]. Global transposon mutagenesis in *M. genitalium* has revealed that approximately 382 of the 482 protein-encoding genes are essential and *obg* is one of them [20], [21]. The Obg *ts* mutants of *B. subtilis* and *E. coli* have been studied extensively. In *B. subtilis*, mutation G79E in the type-II helix d and D84N in the N-terminal Obg domain were shown to be the contributing factor to temperature sensitivity [7]. Analysis of experimentally determined 3D structure of *B. subtilis* Obg has further proven that G79E is the dominant mutation in *ts* phenotype probably by destabilizing the Obg fold at non-permissive temperature, although D84N mutation could further weaken the helix d [22]. Similarly, mutations in *E. coli* ObgE were introduced by site-directed mutagenesis producing mutant ObgE with G80E and D85N substitutions. This mutant ObgE could not support growth at non-permissive temperature of 42°C [24]. Another *E. coli* strain with point mutation S314P in the conserved GTPase G5 motif (Figure 2) of ObgEts showed severe growth defects at 42°C [16], [24]. These findings suggest that the N-terminal domain of Obg proteins has a crucial role in their function.

The Obg glycine residue Gly123Arg, like many other glycine residues in type-II helices a–f within Obg domain, is highly conserved in Obg of various organisms, from human to prokaryotes, indicating its significance in the structure and function of this protein. Other glycine-rich loop-containing proteins, such as CtsR, have also been shown to act as a thermosensor in *B. subtilis*. Specific intrinsic heat-sensor ability of CtsR, a transcriptional regulator, depends on a highly conserved tetraglycine loop within winged HTH domain [52]. The mutation in helix e of MS-H Obg, and more importantly, the reversion of arginine to glycine in the majority of *ts* ^–^ revertants of MS-H implicate its role in temperature sensitivity. Given the essential role of Obg in bacterial cell growth and observed correlation of Gly123Arg mutation in MS-H Obg with a temperature-sensitive phenotype, it would be interesting to explore the role of Obg in mycoplasmas in general and *M. synoviae* in particular. The role of proteins like Obg may be very critical in the biology of mycoplasma-like organisms with a minimal genome and minimal metabolic capacity.

The SOLiD technology used for sequencing in this study involves short sequencing reads. Therefore it is possible that mutations in genes with multiple copies or repetitive elements may have gone undetected. However, only one multigene family (*vlhA*) has been identified in *M. synoviae* genome, in which only one member is associated with a promoter region; the other copies are pseudogenes [53]. The entire sequence of the *vlhA* gene locus (including pseudogenes) was not identified in this study but the sequence of the expressed *vlhA* gene and its promoter was identified and found to contain no difference in the conserved region (results not shown). Nevertheless, it is still possible that other mutations in other genes may have been involved in MS-H temperature sensitivity and/or attenuation.

Experimental proof using complementation of MS-H with the wild-type Obg would be desirable to confirm the role of SNPs found in MS-H Obg in temperature sensitivity and/or attenuation. Given that there is no published methodology for transformation of *M. synoviae* to date, further studies will be essential to establish transformation tools for *M. synoviae* to complement MS-H with the wild-type Obg and study the effects of SNPs, found in this study, on temperature sensitivity and pathogenicity.

## Supporting Information

Table S1 **The origin,** ***ts*** **phenotype and** ***obg*** **genotype of** ***M. synoviae*** **strains/isolates used in this study.**
(DOCX)

## References

[1] Bourne HR, Sanders DA, McCormick F (1990) The GTPase superfamily: A conserved switch for diverse cell functions. Nature 348: 125–132. 2122258 10.1038/348125a0

[2] Bourne HR, Sanders DA, McCormick F (1991) The GTPase superfamily: Conserved structure and molecular mechanism. Nature 349: 117–127. 1898771 10.1038/349117a0

[3] Verstraeten N, Fauvart M, Versées W, Michiels J (2011) The Universally Conserved Prokaryotic GTPases. Microbiol Mol Biol Rev 75: 507–542. 21885683 10.1128/MMBR.00009-11 PMC3165542

[4] Trach K, Hoch JA (1989) The *Bacillus subtilis spo0B* stage 0 sporulation operon encodes an essential GTP-binding protein. J Bacteriol 171: 1362–1371. 2537815 10.1128/jb.171.3.1362-1371.1989 PMC209754

[5] Leipe DD, Wolf YI, Koonin EV, Aravind L (2002) Classification and evolution of P-loop GTPases and related ATPases. J Mol Biol 317: 41–72. 11916378 10.1006/jmbi.2001.5378

[6] Czyz A, Wegrzyn G (2005) The Obg subfamily of bacterial GTP-binding proteins: essential proteins of largely unknown functions that are evolutionarily conserved from bacteria to humans. Acta Biochim Pol 52: 35–43. 15827604

[7] Kok J, Trach KA, Hoch JA (1994) Effects on *Bacillus subtilis* of a conditional lethal mutation in the essential GTP-Binding protein Obg. J Bacteriol 176: 7155–7160. 7961486 10.1128/jb.176.23.7155-7160.1994 PMC197102

[8] Foti JJ, Persky NS, Ferullo DJ, Lovett ST (2007) Chromosome segregation control by Escherichia coli ObgE GTPase. Mol Microbiol 65: 569–581. 17578452 10.1111/j.1365-2958.2007.05811.x

[9] Vidwans SJ, Ireton K, Grossman AD (1995) Possible role for the essential GTP-binding protein Obg in regulating the initiation of sporulation in *Bacillus subtilis* . J Bacteriol 177: 3308–3311. 7768831 10.1128/jb.177.11.3308-3311.1995 PMC177024

[10] Britton RA (2009) Role of GTPases in Bacterial Ribosome Assembly. Annu Rev Microbiol 63: 155–176. 19575570 10.1146/annurev.micro.091208.073225

[11] Scott JM, Haldenwang WG (1999) Obg, an essential GTP binding protein of *Bacillus subtilis*, is necessary for stress activation of transcription factor sigma B. J Bacteriol. 181: 4653–4660. 10.1128/jb.181.15.4653-4660.1999 PMC103599 10419966

[12] Scott JM, Ju J, Mitchell T, Haldenwang WG (2000) The *Bacillus subtilis* GTP binding protein Obg and regulators of the ςB stress response transcription factor cofractionate with ribosomes. J Bacteriol 182: 2771–2777. 10781545 10.1128/jb.182.10.2771-2777.2000 PMC101985

[13] Datta K, Skidmore JM, Pu K, Maddock JR (2004) The *Caulobacter crescentus* GTPase CgtAC is required for progression through the cell cycle and for maintaining 50S ribosomal subunit levels. Mol Microbiol 54: 1379–1392. 15554976 10.1111/j.1365-2958.2004.04354.x

[14] Jiang M, Datta K, Walker A, Strahler J, Bagamasbad P, et al (2006) The *Escherichia coli* GTPase CgtAE is involved in late steps of large ribosome assembly. J Bacteriol 188: 6757–6770. 16980477 10.1128/JB.00444-06 PMC1595513

[15] Lin B, Thayer DA, Maddock JR (2004) The *Caulobacter crescentus* CgtAC Protein Cosediments with the Free 50 S Ribosomal Subunit. J Bacteriol 186: 481–489. 14702318 10.1128/JB.186.2.481-489.2004 PMC305748

[16] Sato A, Kobayashi G, Hayashi H, Yoshida H, Wada A, et al (2005) The GTP binding protein Obg homolog ObgE is involved in ribosome maturation. Genes Cells 10: 393–408. 15836769 10.1111/j.1365-2443.2005.00851.x

[17] Sikora AE, Zielke R, Datta K, Maddock JR (2006) The *Vibrio harveyi* GTPase CgtAv is essential and is associated with the 50 S ribosomal subunit. J Bacteriol 188: 1205–1210. 16428430 10.1128/JB.188.3.1205-1210.2006 PMC1347350

[18] Tan J, Jakob U, Bardwell JCA (2002) Overexpression of two different GTPases rescues a null mutation in a heat-induced rRNA methyltransferase. J Bacteriol 184: 2692–2698. 11976298 10.1128/JB.184.10.2692-2698.2002 PMC135011

[19] Wout P, Pu K, Sullivan SM, Reese V, Zhou S, et al (2004) The *Escherichia coli* GTPase CgtAE cofractionates with the 50 S ribosomal subunit and interacts with SpoT, a ppGpp synthetase/hydrolase. J Bacteriol 186: 5249–5257. 15292126 10.1128/JB.186.16.5249-5257.2004 PMC490892

[20] Glass JI, Assad-Garcia N, Alperovich N, Yooseph S, Lewis MR, et al (2006) Essential genes of a minimal bacterium. Proc Natl Acad Sci USA 103: 425–430. 16407165 10.1073/pnas.0510013103 PMC1324956

[21] Hutchison CA, Peterson SN, Gill SR, Cline RT, White O, et al (1999) Global transposon mutagenesis and a minimal Mycoplasma genome. Science 286: 2165–2168. 10591650 10.1126/science.286.5447.2165

[22] Buglino J, Shen V, Hakimian P, Lima CD (2002) Structural and biochemical analysis of the Obg GTP binding protein. Structure 10: 1581–1592. 12429099 10.1016/s0969-2126(02)00882-1

[23] Kukimoto-Niino M, Murayama K, Inoue M, Terada T, Tame JRH, et al (2004) Crystal Structure of the GTP-binding Protein Obg from *Thermus thermophilus* HB8. J Mol Biol 337: 761–770. 15019792 10.1016/j.jmb.2004.01.047

[24] Kobayashi G, Moriya S, Wada C (2001) Deficiency of essential GTP-binding protein ObgE in *Escherichia coli* inhibits chromosome partition. Mol Microbiol 41: 1037–1051. 11555285 10.1046/j.1365-2958.2001.02574.x

[25] Takeshi H, Niho M, Yuya S, Akatsuki S, Takumi S, et al (2009) Analysis of antibody response by temperature-sensitive measles vaccine strain in the cotton rat model. Comp Immunol Microbiol Infect Dis 32: 395–406. 18249443 10.1016/j.cimid.2007.11.011

[26] Zhongying C, Amy A, George K, Hong J (2008) Molecular studies of temperature-sensitive replication of the cold-adapted B/Ann Arbor/1/66, the master donor virus for live attenuated influenza FluMist® vaccines. Virology 380: 354–362. 18804834 10.1016/j.virol.2008.08.010

[27] Jackwood MW, Saif YM (1985) Efficacy of a commercial turkey coryza vaccine (Art-Vax™) in turkey poults. Avian Dis 29: 1130–1139. 3833219

[28] Morrow CJ, Markham JF, Whithear KG (1998) Production of temperature-sensitive clones of *Mycoplasma synoviae* for evaluation as live vaccines. Avian Dis 42: 667–670. 9876833

[29] Shil PK, Kanci A, Browning GF, Marenda MS, Noormohammadi AH, et al (2011) GapA(+) *Mycoplasma gallisepticum* ts-11 has improved vaccine characteristics. Microbiology 157: 1740–1749. 21310786 10.1099/mic.0.046789-0

[30] Cruz-Vera LR, Toledo I, Hernández-Sánchez J, Guarneros G (2000) Molecular basis for the temperature sensitivity of *Escherichia coli pth*(Ts). J Bacteriol 182: 1523–1528. 10692356 10.1128/jb.182.6.1523-1528.2000 PMC94448

[31] Shahid MA, Ghorashi SA, Agnew-Crumpton R, Markham PF, Marenda MS, et al (2013) Combination of differential growth at two different temperatures with a quantitative real time PCR to determine temperature-sensitive phenotype of *Mycoplasma synoviae* . Avian Pathol 42: 185–191. 23581447 10.1080/03079457.2013.779363

[32] Worth CL, Burke DF, Blundell TL (2007) Estimating the effects of single nucleotide polymorphisms on protein structure: how good are we at identifying likely disease associated mutations? Proceedings of Molecular Interactions: 11–26.

[33] Yousin S, Jan V (2005) SNP discovery in associating genetic variation with human disease phenotypes. Mutat Res 573: 41–53. 15829236 10.1016/j.mrfmmm.2005.01.005

[34] Markham JF, Scott PC, Whithear KG (1998) Field evaluation of the safety and efficacy of a temperature-sensitive *Mycoplasma synoviae* live vaccine. Avian Dis 42: 682–689. 9876836

[35] Drummond A, Ashton B, Cheung M, Heled J, Kearse M, et al. (2011) Geneious v5.5.6. Available: http://www.geneious.com/. Accessed 2011 Dec.

[36] Arnold K, Bordoli L, Kopp J, Schwede T (2006) The SWISS-MODEL workspace: a web-based environment for protein structure homology modelling. Bioinformatics 22: 195–201. 16301204 10.1093/bioinformatics/bti770

[37] Laskowski RA, MacArthur MW, Moss DS, Thornton JM (1993) PROCHECK: a program to check the stereochemical quality of protein structures. J Appl Crystallogr 26: 283–291.

[38] Zhou H, Zhou Y (2002) Distance-scaled, finite ideal-gas reference state improves structure-derived potentials of mean force for structure selection and stability prediction. Protein Sci 11: 2714–2726. 12381853 10.1110/ps.0217002 PMC2373736

[39] Benkert P, Tosatto SCE, Schomburg D (2008) QMEAN: A comprehensive scoring function for model quality assessment. Proteins 71: 261–277. 17932912 10.1002/prot.21715

[40] Eisenberg D, Lüthy R, Bowie JU (1997) VERIFY3D: assessment of protein models with three-dimensional profiles. Methods Enzymol 277: 396–404. 9379925 10.1016/s0076-6879(97)77022-8

[41] Colovos C, Yeates TO (1993) Verification of protein structures: Patterns of nonbonded atomic interactions. Protein Sci 2: 1511–1519. 8401235 10.1002/pro.5560020916 PMC2142462

[42] Worth CL, Preissner R, Blundell TL (2011) SDM–a server for predicting effects of mutations on protein stability and malfunction. Nucleic Acids Res 39: W215–W222. 21593128 10.1093/nar/gkr363 PMC3125769

[43] Schwede T, Kopp J, Guex N, Peitsch MC (2003) SWISS-MODEL: an automated protein homology-modeling server. Nucleic Acids Res 31: 3381–3385. 12824332 10.1093/nar/gkg520 PMC168927

[44] Bhattacharya A, Tejero R, Montelione GT (2007) Evaluating protein structures determined by structural genomics consortia. Proteins 66: 778–795. 17186527 10.1002/prot.21165

[45] Benkert P, Biasini M, Schwede T (2011) Toward the estimation of the absolute quality of individual protein structure models. Bioinformatics 27: 343–350. 21134891 10.1093/bioinformatics/btq662 PMC3031035

[46] Benkert P, Künzli M, Schwede T (2009) QMEAN server for protein model quality estimation. Nucleic Acids Res 37: W510–W514. 19429685 10.1093/nar/gkp322 PMC2703985

[47] Topham CM, Srinivasan N, Blundell TL (1997) Prediction of the stability of protein mutants based on structural environment-dependent amino acid substitution and propensity tables. Protein Eng 10: 7–21. 9051729 10.1093/protein/10.1.7

[48] Worth CL, Bickerton GRJ, Schreyer A, Forman JR, Cheng TMK, et al (2007) A structural bioinformatics approach to the analysis of nonsynonymous single nucleotide polymorphisms (nsSNPs) and their relation to disease. J Bioinform Comput Biol 5: 1297–1318. 18172930 10.1142/s0219720007003120

[49] Mohri Y, Goto S, Nakahigashi K, Inokuchi H (2003) tRNA2Thr complements temperature sensitivity caused by null mutations in the *htrB* gene in *Escherichia coli* . J Bacteriol 185: 1726–1729. 12591892 10.1128/JB.185.5.1726-1729.2003 PMC148054

[50] Nakayashiki T, Inokuchi H (1998) Novel temperature-sensitive mutants of *Escherichia coli* that are unable to grow in the absence of wild-type tRNA6Leu. J Bacteriol 180: 2931–2935. 9603884 10.1128/jb.180.11.2931-2935.1998 PMC107261

[51] Rokop ME, Grossman AD (2009) Intragenic and extragenic suppressors of temperature-sensitive mutations in the replication initiation genes *dnaD* and *dnaB* of *Bacillus subtilis* . PLoS ONE 4: e6774. 19707554 10.1371/journal.pone.0006774 PMC2727948

[52] Elsholz AKW, Michalik S, Zühlke D, Hecker M, Gerth U (2010) CtsR, the Gram positive master regulator of protein quality control, feels the heat. The EMBO Journal 29: 3621–3629. 20852588 10.1038/emboj.2010.228 PMC2982754

[53] Noormohammadi AH, Markham PF, Kanci A, Whithear KG, Browning GF (2000) A novel mechanism for control of antigenic variation in the haemagglutinin gene family of *Mycoplasma synoviae* . Mol Microbiol 35: 911–923. 10692167 10.1046/j.1365-2958.2000.01766.x

[54] Guex N, Peitsch MC (1997) SWISS-MODEL and the Swiss-PdbViewer: an environment for comparative protein modeling. Electrophoresis 18: 2714–2723. 9504803 10.1002/elps.1150181505

