# Supplementary material for: Mutations in GTP Binding Protein Obg of Mycoplasma synoviae Vaccine Strain MS-H: Implications in Temperature-Sensitivity Phenotype
Source: PLoS One. 2013 Sep 17;8(9):e73954. doi: 10.1371/journal.pone.0073954 (PMC3775756; doi:10.1371/journal.pone.0073954)
Supplement: Table S1 — The origin, ts phenotype and obg genotype of M. synoviae strains/isolates used in this study. (DOCX) [file pone.0073954.s001.docx]

Table S1. The origin, *ts* phenotype and *obg* genotype of *M. synoviae* strains/isolates used in this study.

| Strains | Origin | Reference | *ts* Phenotype | *obg* genotype | | |
| --- | --- | --- | --- | --- | --- | --- |
|  |  |  |  | 178^a^ | 367 ^a^ | 629 ^a^ |
| MS-H | Vaccine strain derived from 86079/7NS, Australia | (Morrow *et al.*, 1998) | *ts^+^* | T | A | C |
| 86079/7NS | Parent strain of MS-H vaccine, Australia | (Morrow *et al.*, 1998) | *ts^–^* | T | G | C |
| MS-H^4^ | MS-H-vaccinated flock, Australia | (Markham *et al.*, 1998) | *ts^–^* | T | G | C |
| MS-H^5^ | MS-H-vaccinated flock, Australia | (Markham *et al.*, 1998) | *ts^–^* | T | G | C |
| 93205/1-2a | MS-H-vaccinated flock, Australia | (Markham *et al.*, 1998) | *ts^+^* | T | A | C |
| 93198/6-5b | MS-H-vaccinated flock, Australia | (Markham *et al.*, 1998) | *ts^+^* | T | A | C |
| 94036/6-3a | MS-H-vaccinated flock, Australia | (Markham *et al.*, 1998) | *ts^–^* | T | G | C |
| 93198/6-1a | MS-H-vaccinated flock, Australia | (Markham *et al.*, 1998) | *ts^–^* | T | A | T |
| 94036/10-5a | MS-H-vaccinated flock, Australia | (Markham *et al.*, 1998) | *ts^–^* | T | G | C |
| 93198/5-10a | MS-H-vaccinated flock, Australia | (Markham *et al.*, 1998) | *ts^+^* | T | A | C |
| 93198/1-24b | MS-H-vaccinated flock, Australia | (Markham *et al.*, 1998) | *ts^–^* | T | A | T |
| 93205/2-9a | MS-H-vaccinated flock, Australia | (Markham *et al.*, 1998) | *ts^–^* | T | G | C |
| 94036/2-1a | MS-H-vaccinated flock, Australia | (Markham *et al.*, 1998) | *ts^+^* | T | A | T |
| 93198/4-19a | MS-H-vaccinated flock, Australia | (Markham *et al.*, 1998) | *ts^+^* | T | A | C |
| 94036/2-2a | MS-H-vaccinated flock, Australia | (Markham *et al.*, 1998) | *ts^+^* | C | A | C |
| 93198/3-13b | MS-H-vaccinated flock, Australia | (Markham *et al.*, 1998) | *ts^+^* | T | A | C |
| 93198/3-15a | MS-H-vaccinated flock, Australia | (Markham *et al.*, 1998) | *ts^+^* | T | A | C |
| 93205/2-13a | MS-H-vaccinated flock, Australia | (Markham *et al.*, 1998) | *ts^–^* | T | G | C |
| 93205/9-3a | MS-H-vaccinated flock, Australia | (Markham *et al.*, 1998) | *ts^+^* | T | A | C |
| 93205/8-9c | MS-H-vaccinated flock, Australia | (Markham *et al.*, 1998) | *ts^–^* | T | G | C |
| 93205/10-13a | MS-H-vaccinated flock, Australia | (Markham *et al.*, 1998) | *ts^–^* | T | G | C |
| 94036/9-2a | MS-H-vaccinated flock, Australia | (Markham *et al.*, 1998) | *ts^–^* | T | A | T |

^a^, Location of SNP in *obg* gene
